# Development and Integration of Machine Learning Algorithm to Identify Peripheral Arterial Disease: Multistakeholder Qualitative Study

**DOI:** 10.2196/43963

**Published:** 2023-09-21

**Authors:** Sabrina M Wang, H D Jeffry Hogg, Devdutta Sangvai, Manesh R Patel, E Hope Weissler, Katherine C Kellogg, William Ratliff, Suresh Balu, Mark Sendak

**Affiliations:** 1 Duke University School of Medicine Durham, NC United States; 2 Population Health Science Institute, Faculty of Medical Sciences, Newcastle University Newcastle upon Tyne United Kingdom; 3 Newcastle Eye Centre, Royal Victoria Infirmary Newcastle upon Tyne United Kingdom; 4 Population Health Management Duke Health Durham, NC United States; 5 Department of Cardiology Duke University Durham, NC United States; 6 Department of Vascular Surgery Duke University Durham, NC United States; 7 MIT Sloan School of Management Cambridge, MA United States; 8 Duke Institute for Health Innovation Durham, NC United States

**Keywords:** machine learning, implementation, integration, support, quality, peripheral arterial disease, algorithm, efficacy, structure, barrier, clinical, engagement, development, translation, detection

## Abstract

**Background:**

Machine learning (ML)–driven clinical decision support (CDS) continues to draw wide interest and investment as a means of improving care quality and value, despite mixed real-world implementation outcomes.

**Objective:**

This study aimed to explore the factors that influence the integration of a peripheral arterial disease (PAD) identification algorithm to implement timely guideline-based care.

**Methods:**

A total of 12 semistructured interviews were conducted with individuals from 3 stakeholder groups during the first 4 weeks of integration of an ML-driven CDS. The stakeholder groups included technical, administrative, and clinical members of the team interacting with the ML-driven CDS. The ML-driven CDS identified patients with a high probability of having PAD, and these patients were then reviewed by an interdisciplinary team that developed a recommended action plan and sent recommendations to the patient’s primary care provider. Pseudonymized transcripts were coded, and thematic analysis was conducted by a multidisciplinary research team.

**Results:**

Three themes were identified: positive factors translating in silico performance to real-world efficacy, organizational factors and data structure factors affecting clinical impact, and potential challenges to advancing equity. Our study found that the factors that led to successful translation of in silico algorithm performance to real-world impact were largely nontechnical, given adequate efficacy in retrospective validation, including strong clinical leadership, trustworthy workflows, early consideration of end-user needs, and ensuring that the CDS addresses an actionable problem. Negative factors of integration included failure to incorporate the on-the-ground context, the lack of feedback loops, and data silos limiting the ML-driven CDS. The success criteria for each stakeholder group were also characterized to better understand how teams work together to integrate ML-driven CDS and to understand the varying needs across stakeholder groups.

**Conclusions:**

Longitudinal and multidisciplinary stakeholder engagement in the development and integration of ML-driven CDS underpins its effective translation into real-world care. Although previous studies have focused on the technical elements of ML-driven CDS, our study demonstrates the importance of including administrative and operational leaders as well as an early consideration of clinicians’ needs. Seeing how different stakeholder groups have this more holistic perspective also permits more effective detection of context-driven health care inequities, which are uncovered or exacerbated via ML-driven CDS integration through structural and organizational challenges. Many of the solutions to these inequities lie outside the scope of ML and require coordinated systematic solutions for mitigation to help reduce disparities in the care of patients with PAD.

## Introduction

### Background

The development of machine learning (ML)–driven clinical decision support (CDS) has been rising sharply in the last decade [1,2]. These tools aim to improve health care delivery by enhancing medical decisions with targeted clinical knowledge, patient information, or other information that clinicians can use in conjunction with their own medical knowledge and expertise [3]. Although CDS can improve quality of care and lower health costs, many integrations fail. Thus far, research has found that CDS is most effective at improving health care process (rather than outcome) measures, such as improving the ordering of preventive and treatment services or increasing user knowledge about a medical condition. However, the efficacy of CDS in reducing clinician workload and improving clinical and economic outcomes is more mixed [4-6]. Significant barriers remain in the successful integration of CDS [7,8].

Importantly, much of the clinical ML literature is dominated by in silico algorithm accuracy research [9], with comparatively few studies focusing on real-world integrations that affect patient care [10]. A systematic review of CDS systems found that more than one-third of the systems did not improve clinical practice when implemented [11]. Understanding of translational barriers is limited by the scarcity of research that goes beyond a quantitative assessment of algorithm effectiveness [12]. Qualitative investigations of algorithm integration after successful in silico performance are needed to develop a theoretical understanding of the factors that influence integration failure and success; however, few are available [12-14].

ML-driven CDS is increasingly recognized as being part of a larger sociotechnical system, which includes not only the technology but also the complex social and organizational structures within which the technology is integrated [15]. This interaction between technology and social structure involves many different teams and stakeholders, but only 30% of the scarce qualitative data concerning CDS come from stakeholders besides clinicians (eg, regulators, managers, and developers) [16].

### Objectives

This study aimed to explore the factors that stakeholder groups perceive to influence the integration of ML-driven CDS and to improve our understanding of the needs and aspirations of a wider sample of stakeholders involved in the development and integration of ML-driven CDS. The study was motivated by the need to better understand diverse stakeholder needs to improve algorithm documentation, communication, and training materials, which are crucial for the safe and responsible use of ML-driven CDS [17,18]. In this study, our stakeholder groups were clinical, operational, and technical in nature, and the tool we considered was an algorithm used to identify symptomatic, asymptomatic, and inadequately treated peripheral arterial disease (PAD), to facilitate timely guideline-based PAD care. An additional goal for the integration of ML-driven CDS was to improve equity in PAD management.

Our primary objective was to determine the factors that influence the integration of ML-driven CDS to identify patients with PAD more effectively and equitably for intervention. Our secondary objective was to evaluate the goals and aspirations of ML-driven CDS across stakeholder groups.

## Methods

### Setting

This study analyzes the integration of a previously developed and validated algorithm to identify patients with PAD using revascularization procedure data, encounter diagnoses, and clinician specialty information for encounter programs [19]. The algorithm was originally developed by an interdisciplinary team as a part of a research project. In April 2021, senior leaders at a single academic medical network, an integrated care system of community and specialist health care providers, tasked the affiliated Institute for Health Innovation (IHI) to implement the algorithm in collaboration with the regional Population Health Management Office (PHMO). IHI is an organization focused on developing and implementing impactful health and health care innovations, whereas the PHMO is an entity that manages value-based contracts and care management programs.

The IHI provided project management and technical support for ML-driven CDS integration. The PHMO provided administrative and operational oversight as well as a pharmacist to help with medication prescription interventions. Cardiologists and vascular surgeons were the initial target users of ML-driven CDS. The PAD algorithm had already undergone in silico validation using retrospective data, demonstrating the validity and potential utility of the algorithm. Prospective validation of the algorithm was conducted in a separate study and will be presented in future work. The primary goal of the ML-driven CDS integration was to identify patients with progressing PAD for intervention by their primary care provider (PCP) to prevent downstream complications, including cardiovascular and limb events (eg, amputations). In addition, health system leaders sought to improve equity in the treatment of PAD and decrease disparities in limb and cardiovascular complications.

### PAD Algorithm and Workflow

PAD affects approximately 10 million Americans but remains significantly underdiagnosed in primary care settings [20]. Diagnosis and procedure data are often missing, making it challenging to identify patients with PAD for disease management programs [21,22]. Care is also fragmented across many different specialties (ie, primary care, cardiology, vascular surgery, and podiatry), leading to confusion among patients and poor coordination of care. Ultimately, many patients with PAD do not benefit from evidence-based interventions [19]. For example, a recent analysis from the American Heart Association’s Statistics Committee [20] reported that only 6% to 18% of patients who underwent limb amputation or died of a complication of PAD were on appropriate statin therapy. In the United States and elsewhere, rates of guideline-directed interventions for PAD, such as statin therapy, have also been reported to be particularly low among minority ethnic groups and patients with limited resources, contributing to disparities in health outcomes [20,23]. This study defines patients with PAD not on statin therapy as being inadequately treated.

**Figure 1:**
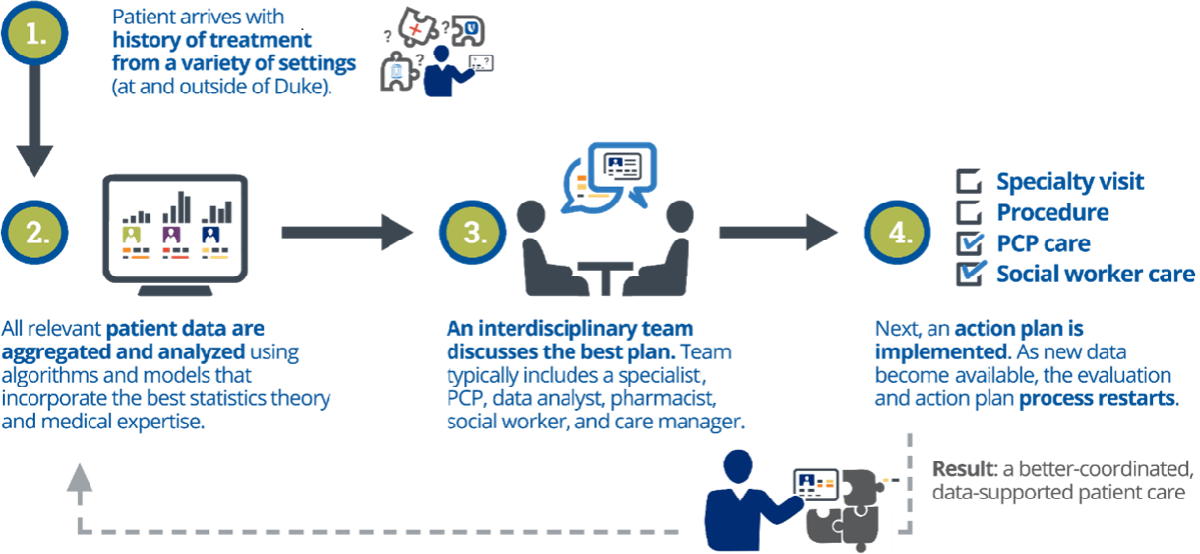
Population Rounding workflow that aggregates data across sites for interdisciplinary teams to review high-risk patient cases and send personalized recommendations to primary care physicians. PCP: primary care provider.

During data collection for this qualitative study, the ML-driven CDS was run weekly on all adult patients with a clinical encounter with a PAD-associated International Classification of Diseases code in the past week within the health institution. Only patients within the institution’s care management program were included as part of this study and represented approximately two-thirds of the weekly ML-driven CDS output. The care management program includes >100,000 patients at this institution and is inclusive of a variety of private and public insurance plans, including Medicare, Medicare Advantage, Medicaid, and employee health plans, as well as the enrollment of underinsured or uninsured patients. More than 1000 patients were identified each week, and the medical charts of these patients were reviewed by an interdisciplinary team in a weekly process called Population Rounding (trademarked by Duke Health). During this pilot phase, patients with upcoming PCP appointments were prioritized. This approach aimed to increase the saliency of communication for PCPs, as they prepared for patient appointments to increase the probability of PCP intervention. After preliminary screening, approximately 20 out of 30 selected patients were reviewed in the rounding discussions, which took place for 60 minutes via Zoom (Zoom Video Communications). As described in the original paper [21], PAD was confirmed using the ankle-brachial index, and a history of prior revascularization or lower-extremity amputation was used to indicate symptomatic PAD. For each patient, the interdisciplinary team identified potential gaps in care, especially in preventive interventions such as statin use, smoking cessation, or additional specialty care. If the team agreed on specific treatment recommendations, a personalized message was sent through the electronic health record (EHR) to the patients’ PCP. No best-practice alerts or other pop-up notifications were used. Of the patients presented during the rounds, approximately two-thirds of patients discussed received an intervention. This Population Rounding model (Figure 1) had been used previously by the PHMO for chronic kidney disease and other chronic conditions [24] and was developed with inspiration from the telemedical ward model in the United Kingdom [25] as well as more traditional care management programs in the United States [26]. The Population Rounding model is novel in its ability to integrate a predictive model to identify patients, interdisciplinary rounding sessions, and the ability to make referrals and enroll patients in identified population health programs.

### Study Design and Data Collection

This study was conducted in accordance with the COREQ (Consolidated Criteria for Reporting Qualitative Research) checklist (Multimedia Appendix 1) [27]. All 12 stakeholders directly involved in the algorithm integration were interviewed using a videoconferencing platform. All invitations to participate in the study were accepted. Of the 12 participants, 5 (42%) were from the technology team, 5 (42%) were from the operations team, and 2 (17%) were from clinical end users. The individual roles included within each stakeholder group are described in Table 1.

Semistructured interview guides were developed for the technical, operational, and clinical stakeholder groups (Multimedia Appendix 2). To address the primary aim of this study, questions for each participant explored the factors influencing the integration of a PAD identification algorithm to implement timely guideline-based care. To address the secondary aim of this research, questions targeting personal success criteria for integration were asked to each participant.

**Table 1:** Stakeholders interviewed.

Stakeholder group	Included roles	Stakeholder, n (%)
Clinical end users	Vascular surgeon and primary care provider	2 (17)
Health systems operations (PHMO^a^)	Administrative leader, nurse, operational leader, and clinical pharmacist	5 (42)
Health technology team	Program manager, data engineer, and data scientist	5 (42)

^a^PHMO: Population Health Management Office.

Interviews lasting 45 to 60 minutes were conducted by SMW, a medical undergraduate and public health postgraduate, and HDJH, an ophthalmology resident and implementation science doctoral student. Besides the participants, nobody else was present for the interviews, and no repeat interviews took place. Audio recordings were pseudonymized and digitally transcribed to create field notes. SMW and HDJH are formally trained in qualitative research methods and practice clinically in the United States and the United Kingdom, respectively. The interviewers were introduced to each participant before and at the outset of each interview. Neither SMW nor HDJH had relationships with the team implementing the PAD algorithm that preceded the project. To foster consistency of approach, the first 5 interviews were conducted by SMW and HDJH together, with the later 7 interviews completed by SMW or HDJH alone. The Nonadoption, Abandonment, and Challenges to the Scale-Up, Spread, and Sustainability framework and iterative review by the multidisciplinary authorship team informed the development of interview guides. The Nonadoption, Abandonment, and Challenges to the Scale-Up, Spread, and Sustainability framework was selected because it outlines technology implementation factors at the policy, organizational, and practice levels [28]. Participants consented to the audio recording, were informed about the confidentiality of their responses, and received no compensation. Data saturation did not affect the sampling approach [29]. This was because the pilot stage of integration at which interviews took place meant that only a relatively small group of stakeholders had interacted with the algorithm, and therefore, interviewing them all was feasible.

### Data Analysis

An inductive approach was used for data analysis [30]. The interviewers (SMW and HDJH) reviewed the digital transcriptions along with audio recordings to ensure accuracy and begin data familiarization. Two members of the team (SMW and HDJH) coded the first 4 transcripts independently and then met to discuss their independent approaches and cocreate a single codebook of higher-order categories (eg, health care workforce), containing several codes (eg, available skill sets and disciplines; Multimedia Appendix 3). The new codebook was used independently by both researchers for the remaining interviews and was updated iteratively through consensus, where data fell outside the present codes. NVivo 12.0 (QSR International Pty Ltd) facilitated the coding and analysis process.

Following the coding process, SMW and HDJH engaged in an initial thematic analysis independently at first and then together [30]. This initial thematic analysis was then shared and revised collectively with additional coauthors to assess thematic saturation and draw on various academic, technical, clinical, and operational perspectives. Thematic saturation was determined to be met after an initial analysis with 10 interviews, and no further interviews were required [31]. Findings were not directly fed back to the participants but will support the ongoing implementation of the PAD algorithm and other ML-driven CDS.

### Ethical Considerations

As per the institution’s Health Institutional Review Board Quality Improvement definition and protocols, this study met the criteria for quality improvement, and the study was not submitted for ethical approval by the institutional review board (Multimedia Appendix 4). Although implicit written consent was received through email exchanges before each interview, standardized consent forms were not used. Participants provided informed consent through review of the participant information sheet (Multimedia Appendix 5), agreement to provide time for the interview, and agreement to have the interview recorded.

## Results

### Overview

The analysis revealed 2 themes related to the factors that influence the integration of a PAD identification algorithm to implement timely care and 1 theme related to equitable guideline-based care: (1) positive factors translating in silico performance to real-world efficacy, (2) organizational factors and data structure factors affecting clinical impact, and (3) potential challenges to advancing equity with ML.

### Theme 1: Positive Factors Translating In Silico Performance to Real-World Efficacy

#### Overview

This theme is related to the successful translation of an ML-driven CDS validated in silico to create a real-world clinical impact. The following four positive factors were identified: (1) clinical leadership, (2) trustworthy workflows, (3) early consideration of end-user needs, and (4) targeting an actionable problem. Each factor is described in the following sections, and representative quotations are presented in Textbox 1.

**Positive factors translating in silico algorithm performance to real-world efficacy.**

**Early consideration of end-user needs**

- “The first set of impacts are the hope that primary care physicians find these recommendations useful. And maybe they don’t adopt all of them. But they read all of them. And they consider all of them and then make a determination based on that recommendation... so with population health, always residing first with the primary care physician, or anchoring, first around the primary care physician.” [Operational stakeholder]
- “I think the big learnings from PAD are probably going to be how to represent this data, like how to build out that dashboard for consumption, that’ll be a lot of back and forth with the stakeholders that are going to be using it. They don’t want clinicians to have to go to a separate tool to consume model results. They don’t want to have to open another dashboard that should be in Epic already.” [Technical stakeholder]
- “We’ll have to navigate through that [physician receptiveness] and really understand how our clinicians want to receive this information about things that they might want to consider doing differently with their patients.” [Operational stakeholder]
- “You want to focus on the patient in the room and not all the freaking alerts that you’re trying to excuse. It’s very distracting... I don’t know what the right answer is or how to make it user friendly, but to keep the patient first and foremost, and they’re in my room, they get my attention. Not the computer screen, right?” [Clinical stakeholder]
- “I hate BPA’s [Best Practice Alerts] or they pop up in EPIC charts three times- I just close them so their utility is gone. But anyway, to be thoughtful about how to design the way they interact with us and remind because I know they’re trying to help our practice, so I want the help.” [Clinical stakeholder]

**Targeting an actionable problem**

- “I mean, it [the algorithm] has to solve a real problem. Like, I’m not interested in models that in a clinical sense, identify data that I could just identify in the course of my daily work. I don’t need a model to pull out someone’s, you know, this, that or the other risk, if that risk is either otherwise obvious or not actionable... So that’s one major thing is it has to have a use to it that was generated with clinicians in mind, in partnership with physicians, and also it has to be shown to actually work.” [Clinical stakeholder]
- “From a model standpoint, how do we get the workflow set up to start helping patients today, really.” [Technical stakeholder]
- “So at a macro level for population health, so far, there seems to be one thing that most strongly contributes...to better health outcomes at a macro level. And that is regular access to primary care. We see that the more primary care that our patients regularly complete or obtain, the better outcomes and better quality, the greater cost efficiency, less avoidable utilization. Just that one behavior, or that one process measure. If we can help primary care practices, keep their patients coming regularly, and support high quality primary care visits, all the many other good things are gonna happen.” [Operational stakeholder]

**Clinical leadership**

- “The difference in [algorithm] performance is negligible compared to the difference that a good physician champion makes, or a good intervention plan makes. Those are by far and away the most important things to the success of a project. The actual model itself is, as much as I might delude myself or whatever, it’s actually not that important.” [Technical stakeholder]
- “Dr XXX [redacted identifier] obviously was very aware, she was kind of the one that spearheaded [the integration] and wanted to get this going. So I think you know, [targeting clinical need] is her role. I’m sure with her specialty, she probably realized that it was an area that needed more attention.” [Operational stakeholder]

**Trustworthy workflows**

- “If you can do like 80% of the accuracy of the best model, but you have a good clinical team who are willing to implement it and to give feedback and to develop interventions that are tailored specifically for the right people. I’ll take that 80% every single time. There’s no question.” [Technical stakeholder]
- “So I personally would probably like to see some citations too, kind of show where it’s [the machine learning (ML)–driven clinical decision support (CDS)] coming from. But if it has made it to the level that IHI and PHMO are considering it, I think probably most clinicians have some faith in the fact that it’s been endorsed and reviewed and kind of is considered standard of practice.” [Clinical stakeholder]
- “I think we figured out, this [workflow] is the way to roll any new specialty into it [using an ML-driven CDS] and we have a very good structure in place. And I think we know like, we know the things we need to identify to kind of get our initial data report, you know that sort of stuff and then, once we have that, just put it on its legs and see if it’s gathering the patients, we need. So I think, because we have so much experience with it, when we want to bring on a new specialty for rounds, it’s not so daunting because we know exactly what we need to do because we’ve been doing it now for a while.” [Operational stakeholder]
- “And we, over the last three or four years have been moving into expanding our competencies and partnerships with community based organisations, first of all screening for and understanding non clinical barriers to health, social barriers to health, but partnering with organisations in the community, who can help address those barriers, if we can coordinate the patient over to that resource.” [Operational stakeholder]

#### Early Consideration of End-User Needs

Almost all technology and operational stakeholders mentioned the importance of the early consideration of end-user needs. Early consideration of end-user needs ensured that there were appropriate data inputs to generate the desired outputs, the ML-driven CDS tool did not create an undue burden of use, and the algorithm was built to prompt specific interventions. The ML-driven CDS tool for PAD has 2 sets of end users: primary users are Population Rounding team members who interact directly with the product to identify interventions and secondary users are the PCPs who receive recommendations from the Population Rounding team. Both user groups were actively considered during the design of the algorithm. For example, because the Population Rounding team members had a high degree of technical literacy, they prioritized the visibility of all relevant data over the simplicity of the user interface. Consequently, a relatively data-heavy dashboard was designed to comprehensively deliver information that the Population Rounding team member leading rounds (the vascular surgeon) might need to drive interdisciplinary discussions and decision-making.

Along these same lines, because the PCPs were already burdened with managing multiple chronic and acute conditions, the workflow was designed such that more work was not created for the PCPs. For example, instead of interrupting a PCP’s clinic visit with alerts or notifications within the EHR, intervention recommendations were sent to the PCP before the patient visit. Notifying a PCP in advance also gives PCPs an opportunity to prepare for conversations with patients.

#### Targeting an Actionable Problem

As ML-driven CDS systems become increasingly prominent in health care, clinical and operational stakeholders emphasized the importance of developing an ML-driven CDS focused on facilitating clinical decision-making around an actionable problem. They expressed frustration that the algorithm outputs associated with many ML-driven systems did not change clinical care or impact outcomes. The technology team similarly echoed that they relied on clinician input to help them design the product in a meaningful way. When technology team members were asked about their vision of success for the project, they cited the importance of algorithm performance but also expressed the desire to see real-world improvements in long-term clinical outcomes.

#### Clinical Leadership

Multiple stakeholders across groups spoke about the importance of strong clinical leadership in both the initial product design process and integration of the final product. Technical team members emphasized that a perfectly performing algorithm would not create the desired outcomes in a clinical setting if there was no strong leadership to help tailor, implement, and provide feedback on the ML-driven CDS:

The difference in performance is negligible compared to the difference that a good physician champion makes, or a good intervention plan makes.

Technical stakeholder

In our case, the same clinical leader who developed the initial algorithm led the Population Rounding sessions with the PHMO. This longitudinal leadership commitment was identified in interviews as a critical factor in successfully transitioning from in silico algorithm performance to the real-world clinical use of the product.

#### Trustworthy Workflows

Participants also emphasized that the workflow in which the algorithm was integrated was more important than the accuracy of the algorithm. In this project, the PHMO Population Rounding workflow was identified as a key factor that enabled project success. This well-established interdisciplinary approach requires significant resources and personnel, whereby the team manually reviews each patient chart identified by the algorithm to tailor recommendations to the PCPs. The synchronous, interdisciplinary nature of Population Rounding allows for rapid communication across teams and allows teams to nimbly respond to obstacles that arise because it includes representatives of different stakeholder groups. For example, PHMO team members can draw on their preexistent community partnerships to manage social barriers to health. Furthermore, including a vascular specialist as the leader of this process lends credibility to the ML-driven CDS and workflow. PCPs who received recommendations downstream from the Population Rounding were familiar with the specialist and the specialist’s expertise in the management of PAD. This specialist’s recognized “stamp of approval” allowed PCPs to trust recommendations without personal familiarity with the ML-driven CDS or relevant performance metrics.

### Theme 2: Organizational and Data Structure Factors Affecting Clinical Impact

#### Overview

The next theme captures the organizational and data structure factors that shape the clinical impact of the PAD algorithm. The following three negative factors were identified: (1) failure to incorporate on-the-ground context, (2) the lack of feedback loops, and (3) data silos. Each factor is described in the following sections, and representative quotations are presented in Textbox 2.

**Organizational factors and data structure factors affecting clinical impact.**

**Failure to incorporate on-the-ground context**

- “This is a really high risk patient like I’m really concerned about this person, but it’s all the things that you can’t pull into an algorithm. Like is this person compliant with their medications? What social drivers are they dealing with? That’s why we think of our provider referrals.” [Operational stakeholder]
- “Yeah, we have a social drivers wheel [theoretical framework describing social determinants of health]. But that wheel is only as good as the number of people who are doing SDH screening and populating the wheel currently.” [Operational stakeholder]

**Lack of feedback loops**

- “You know, I think probably the biggest feedback that can give confidence in the algorithm was when we do get a response from a provider, and they thank us, or we can go back in and we see that the patient got referred to smoking cessation, or that, you know, a suggestion that we gave was actually implemented or was considered.” [Operational stakeholder]

**Data silos**

- “One potential thing that can be difficult for these models is: patients who have a long established medical history versus those that don’t, and differentiating between 1) somebody who doesn’t have a long medical history because they are a newer patient or they have (just) established care at [this institution] versus 2) one who seeks the majority of their care outside of [this institution] and just come to [this institution] for, for example, like an inpatient stay or something like that.” [Technical stakeholder]
- “About 20% of our population does not have data. We support primary care physicians at other practices that use different EMRs, different medical records, okay, and we are blind. So we will be over-selecting and over-serving 80% and under-selecting and under-serving 20% of our population because of a structural dynamic here in the United States. Despite all the investment and health IT there is this effect of don’t share any data with anybody until it’s absolutely necessary.” [Clinical stakeholder]
- “And I should also say those even worse, those 20% [who we don’t have data on], or a higher proportion of them are older, coming from an economically higher social deprivation index, higher disease burden, non-white... Conflict between efficacy of the model and performance and the equity of its distribution or application.” [Operational stakeholder]

#### Failure to Incorporate On-the-Ground Context

Exercise, nutrition, smoking, and medication adherence are critical components of the management of PAD, and clinicians expressed concern about adopting the algorithm to make intervention recommendations without knowing more about these factors. Operational stakeholders acknowledged that even with a highly accurate ML-driven CDS, frontline PCPs had an additional context that was not captured by the algorithm. PCPs understood the barriers faced by individual patients, such as why a patient might be hesitant to try a new medication or why they might not be able to see a specialist more often. The algorithm’s potential impact was limited by the inability to account for the barriers patients faced in managing PAD.

#### Lack of Feedback Loops

Adoption of the algorithm was also limited by the lack of a feedback loop between the actions that users took in response to the interventions recommended, on the one hand, and patient outcomes associated with these actions, on the other hand. After PCPs were sent tailored recommendations from the Population Rounding team, there was no automated feedback loop in place to track patient-level outcomes. Clinical stakeholders were concerned that they lacked visibility on whether interventions recommended to PCPs were acted upon, ignored, or even seen. Clinical stakeholders on the Population Rounding team did not learn whether PCPs had made the recommended change in a patient’s care until the patient’s next review, which often did not occur for many months. Even in cases where the PCP acted on a recommendation provided by the Population Rounding team, there was another layer of unknown patient willingness or ability to adhere. All the stakeholders spoke about the need for better feedback loops for PCPs to share their reasons for not accepting algorithmically shaped recommendations by Population Rounding team members. For example, PCPs shared that they often did not accept Population Rounding team members’ recommendations to promote statin use, as this was particularly challenging for PCPs to do with patients who had a bad experience with the medications in the past.

Operational stakeholders had to manually review a patient’s chart to follow up on the effectiveness of the intervention recommendations. Reviews were planned for every 6 months to assess outcomes. This timing was meant to allow the clinical team to understand if changes were needed to better communicate with PCPs or if the recommendations themselves needed to be changed. Without this type of feedback, opportunities to improve ML-driven CDS and workflow are difficult to identify. Operational stakeholders expressed an interest in developing an internal feedback system that would provide feedback in a way that minimized the additional burden on the PCP. The IHI technical team is now exploring an approach to automatically track PCP order placement for statins or smoking cessation referrals.

#### Data Silos

A key theme discussed by all stakeholder groups was the concern of inequitable outcomes because of the challenges in aggregating data across siloed systems. Specifically, this relates to the lack of documentation and capture of data for patients who either did not receive a substantial amount of care at this institution or whose PCPs used EHRs that did not interface with the institution’s system. The inability to capture and aggregate data for these patients limited the utility of the PAD algorithm. However, patients who received health care across multiple sites or in more rural settings were thought to be more likely to have significant socioeconomic disadvantages.

Although this observation is framed here as a barrier to the impact of the PAD algorithm, it is also a potential source of inequity. Clinical and operational stakeholders recognized that about 20% of patients who did not have EHR data were effectively invisible to the algorithm:

So we will be over-selecting and over-serving 80% and under-selecting and under-serving 20% of our population because of a structural dynamic.

Operational stakeholder

However, clinical and operational stakeholders feared that the algorithm could potentially worsen inequities by not serving patients whose EHRs were inaccessible.

### Theme 3: Potential Challenges to Advancing Equity With ML

#### Overview

The ML-driven CDS for PAD was perceived as a tool that could be used to advance equity and reduce disparities in care. Nevertheless, three challenges to advancing health equity emerged in many interviews: (1) difficulty in identifying impactable patients, (2) missing social context and (3) insurance restrictions. Each challenge is described in the following sections, and the representative quotations are presented in Textbox 3.

**Potential challenges to advancing equity.**

**Difficulty in identifying impactable patients**

- “The one piece... that we’ve not really been able to tap into yet is impact-ability. So you have this cohort of people who would benefit from this service, but what factors of them really make them good candidates to be intervened upon? Sometimes when you’re dealing with chronic complex diseases, when it progresses beyond a certain point, you know, there’s not a lot that you can do to intervene.” [Operational stakeholder]
- “I would think that the model’s design can be internally equitable, but how it’s used or applied might still require that discipline or deliberative consideration of whether the service itself is equitable.” [Technical stakeholder]

**Missing social context**

- “I think we’re really so limited to what’s available in the electronic health record and or claims. So the things we can’t see are social drivers right now.” [Clinical stakeholder]
- “There’s this commonly understood notion that only 20% of our healthcare outcomes are really driven by clinical interventions inside the clinic and the other 80% is a mix of various uncontrollable or controllable factors uncontrollable like background genetics, or split controllable factors related to social drivers of health or the geography of opportunity where people reside.” [Operational stakeholder]
- “This is a group, a subgroup, that certainly has a high burden of social barriers to health that in addition to having ample access to primary care, high quality primary care, the ability of keeping our neighbors connected with the community and the resources is going to be an interesting place for us to grow in to.” [Operational stakeholder]
- “So we can look at race and sex and ethnicity and say, you know, is the model for whatever reason, showing higher you know in one race versus another and one sex versus another. Yeah, we have the ability to do that in post, but I unfortunately don’t really know how the model determines one way or the other, or biases one way or the other. I imagine it ignores it completely, but that’s not always true.” [Technical stakeholder 1]

**Insurance restrictions**

- “However, our contracts, our relationships with payers attribute or designate a set of patients, and put them in a population that they say we are accountable to.” [Operational stakeholder]
- “But if I look across my entire pocket population, and just like that had PAD, they’re not all going to be attributed to one of our value-based care arrangements. So who’s going to be taking care of those patients?” [Operational stakeholder]
- “So like patients who aren’t eligible for [Duke Well], they have trouble kind of intervening on those patients.” [Technical stakeholder 2]

#### Difficulty Prioritizing Impactable Patients

Operational stakeholders discussed the challenge to advancing health equity—wanting to intervene on all identified patients but only having a limited number of hours and resources available per week. The ML-driven CDS tool for PAD can be run on all patients in a population, but only a small number of high-risk patients can be reviewed each week. Stakeholders described the difficult trade-offs they had to make when considering how to select patients to prioritize for Population Rounding. Given that this project was a pilot meant to demonstrate value and secure sustaining investment, clinical and operational stakeholders wanted to prioritize patients who seemed the most “impactable.” Patient “impact-ability” was operationalized by requiring a patient to have a PCP appointment within 1 week of the Population Rounding review. This approach maximized the saliency of communication for PCPs and increased the probability of action as the PCPs prepared for upcoming appointments but limited the algorithm’s impact on patients with minimal access to PCP care. All stakeholder groups recognized the potential need to revise how patients were prioritized for Population Rounding after the pilot period.

#### Missing Social Context

Operational and clinical stakeholders noted that they were conscious of the fact that patients with PAD from historically marginalized populations face significant barriers to care and that the ML-driven CDS tool might be less effective for marginalized patients. These stakeholders recognized that this was an important limitation of the current integration approach. The PAD algorithm uses data routinely captured in the EHR and medical claims and does not incorporate granular information related to social determinants that account for many of the barriers to care. As described above in *Theme 2* (*Failure to Incorporate On-the-Ground Context* subtheme), PCPs who understood individual patients’ social context could augment the algorithm to better tailor interventions. However, gaps in data on social determinants of health limited how well the PHMO could efficiently target social support services. Operational stakeholders saw an opportunity to enhance the ability to advance equity by supplementing the output of the ML-driven CDS tool for PAD with information about the social determinants of health and barriers to care. This could enable tailored medical interventions for PAD to be coupled with social support services to address barriers to accessing care related to the interventions.

#### Insurance Restrictions

Another challenge to advancing health equity is the way in which insurance agreements vary. Only a subset of insurance plans covered the full range of clinical capabilities and resources leveraged by the Population Rounding workflow. For example, some insurance agreements covered care management or pharmacist support, whereas others did not. This disparity in clinical service coverage means that not all patients are eligible for Population Rounding. For the initial pilot period, eligibility was limited to specific insurance plans, thus limiting the potential benefits of the ML-driven CDS tool for PAD. Although the proportion of patients at high risk of PAD ineligible for Population Rounding because of insurance coverage was unknown to interviewees, interviewees expressed concern that not all high-risk patients could benefit from the program. The algorithm can be easily run on tens or hundreds of thousands of patients; however, resource constraints related to insurance status could reinforce inequities in access to care.

### Stakeholder Group Comparison

The secondary aim of this study was to identify differences in project aspirations across stakeholder groups. All interviewees were asked to describe their own vision of success for the ML-driven CDS tool for PAD, and clinical, operational, and technical stakeholders had different aspirations. Short data excerpts are summarized in Textbox 4.

**Differences in stakeholder aspirations.**

**Clinical**

- “Performs well and reproducibly in a contemporary timeframe”
- “Portable to different health systems to facilitate multicenter research or for other centers to identify PAD patients”
- “Less preventable [adverse] events”
- “I definitely need to be more educated and on top of kind of identifying peripheral artery disease”

**Operational**

- “Drive down health care costs”
- “Team using the model is comfortable”
- “PCPs are often, if not always, considering the recommendations”
- “We’re able to work with primary care providers”
- “Identifying impactable patients”
- “Measurable reduction or measurable increase”

**Technical**

- “Minimizing downtime”
- “Streamline the process”
- “Improve efficiency”
- “Data is consumable and useful for clinicians”
- “Built in all the monitoring for the inputs and it’s generating outputs as we expect”

Clinical stakeholders aspired to reduce “preventable” events related to PAD and promote clinical education, research, and dissemination to other sites. Operational stakeholders aspired to reduce health care costs and improve the clinical outcomes. Notably, almost all operational stakeholders hoped that the project would build trust between the PHMO and PCPs and that PCPs would act on recommended interventions. In addition, operational stakeholders hoped that clinical users would become comfortable working with the ML-driven CDS. Finally, technical stakeholders aspired to minimize downtime, streamline the workflow process for clinical users, and improve the overall efficiency of how the data were consumed. Technical stakeholders emphasized the importance of creating an algorithm that generated appropriate outputs and being able to monitor and respond to changes in algorithm behavior.

## Discussion

### Principal Findings

In this study, we conducted interviews with members of the technical, operational, and clinical stakeholder groups to explore the positive and negative factors of the integration of a PAD identification algorithm to implement timely guideline-based care. We advanced insights related to three themes: (1) positive factors of translating in silico ML-driven CDS performance to real-world efficacy, (2) organizational factors and data structure factors affecting clinical impact, and (3) challenges to advancing equity with ML. We also highlighted nuanced differences in goals and aspirations across stakeholder groups to better understand how transdisciplinary teams work together to integrate ML-driven CDS.

This study is unique in several important ways. First, although there are qualitative research studies examining ML integration into clinical care, almost all prior studies have focused on the experience of clinicians [32-34]. One study did incorporate patient perspectives, and another study included clinician and developer perspectives [35,36]. Although there are a few examples examining non–ML-driven CDS integration, to our knowledge, this is the first study to incorporate technical, operational, and clinical stakeholder perspectives on ML-driven CDS integration [37,38]. Given that technology adoption decisions are often made at the organizational leadership level [28], it is critical to understand how administrative and operational leaders perceive the risks and benefits of ML-driven CDS integration.

Second, although there is an extensive and growing body of literature surrounding the potential for bias in ML-driven CDS integrated into clinical care, prior work has focused on the quantitative measurement of bias within algorithms [39,40]. This focus on the algorithm itself results in technical recommendations to minimize bias, such as thoughtfully selecting algorithm outcome labels and scrutinizing algorithm inputs [41,42]. To our knowledge, this is the first study closely examining the structural and organizational, rather than technical, challenges to advancing equity through integration of ML-driven CDS into clinical care. Although there may be opportunities to improve the PAD algorithm, the study surfaced several opportunities to improve the ability of the algorithm to help close disparities in the care experienced by patients with PAD.

Despite a growing consensus about the necessity of understanding ML-driven CDS as a part of larger sociotechnical systems, most ML research in health care continues to focus on the technical aspects of algorithms. The ML-driven CDS is only 1 component of the workflow, and all integrations of ML require close attention to the nontechnical aspects of the workflow from a larger system’s standpoint. Our study found that across stakeholder groups, the factors that led to successful translation of in silico algorithm performance to real-world impact were largely nontechnical, given adequate efficacy in retrospective validation. A technical stakeholder stated the following:

The actual model itself is, as much as I might delude myself or whatever, is actually not that important.

Our findings emphasize that high-quality care and health outcomes are shaped by the adoption of a network of stakeholders and the ways in which they work together [43-45]. Therefore, ML-driven CDS integration must target organizational structures, cultures, and aspirations [46]. Our study showed how this can be successfully accomplished, even when accounting for the needs of primary (clinicians who participated in Population Rounding) and secondary users (PCPs caring for patients with PAD). Our findings build upon recent work on ML integration in the inpatient setting, which revealed the importance of engaging the range of clinicians who provide care for patients in both the early and severe stages of disease [47]. A key strategy that drove alignment was the consistent clinical leadership of a downstream specialist in the conceptualization, planning, and execution phases of the ML-driven CDS project. A technical stakeholder expressed eagerness to compromise on algorithm performance for more engagement from expert clinicians (refer to “Trustworthy workflows” in Textbox 1)*.* However, this suggests that high-resource health care delivery settings that bring together clinical and technical experts to integrate ML-driven CDS are most likely to succeed [48]. In contrast, low-resource settings that do not have ML developers to work hand in hand with clinicians may struggle to successfully integrate ML-driven CDS.

Our study also emphasized the importance of workflow design and providing support in a familiar and trustworthy manner to frontline clinicians. The Population Rounding workflow was described as a critical component of success because it did not increase alert fatigue for PCPs (refer to “Early consideration of end-user needs” in Textbox 1). Pop-up alerts in EHR are often dismissed, do not improve care [49-52], and contribute significantly to physician burnout [53,54]. The personalized interventions sent by the vascular specialist addressed immediate gaps in care and educated PCPs on how to care for other patients at high risk of PAD (refer to “Clinical stakeholder group” in Textbox 4). The Population Rounding workflow also relieved PCPs of the need to understand technical details of the PAD algorithm. Each case was reviewed by an interdisciplinary team, and because the vascular specialist led the communication with PCPs, the recommendations were perceived as trustworthy. This was different from a sepsis algorithm that placed nurses as primary ML-driven CDS users who sent recommendations to more expert secondary users (emergency department physicians) [33]. In this case, although the treatment recommendations were aligned with federal quality measures, physicians often questioned the recommendations, and nurses developed novel approaches to navigate collaborative sepsis management.

Although the PAD algorithm was not visible to PCPs, the ML-driven CDS integration serves as an important example of how ML algorithms can support frontline clinicians. In many contexts, ML serves the interests of organizational leaders to extract value from frontline clinicians [35]. Workers were surveilled, and algorithms were used as tools to influence decision-making and behavior on the front lines. A few characteristics of the PAD system mitigated these concerns. First, the PCPs retained autonomy in making clinical treatment decisions for their patients. Clinical and operational stakeholders valued the role of PCPs because they understood that PCPs knew patients in a way that the ML-driven CDS system could not account for. An operational stakeholder explained that PCPs know best how to treat high-risk patients because of “all the things that you can’t pull into an algorithm.” Second, as described above (Textbox 4), the interests of the PCPs were aligned with the Population Rounding team. The vascular specialist and PCPs worked for the same organization and cared for similar patients. Third, the clinical and operational stakeholders understood that the PAD system could not be effective without strong buy in from the PCPs. A clinical stakeholder described the following:

...the biggest feedback that can give confidence in the algorithm was when we do get a response from a [PCP] provider, and they thank us.

Trust in the algorithm was ultimately tied to how PCPs responded to recommendations sent by the Population Rounding team. This qualitative analysis identified a gap in built-in feedback loops, which was communicated to the technical team for improvement in the future.

Although the ML-driven CDS system for PAD was seen as a potential opportunity to address health disparities, our study highlighted several important challenges to advancing equity through the use of ML. None of the challenges discussed by stakeholders were specific to how the algorithm was built but pertained to data and organizational structures. First, both clinical and operational stakeholders recognized that a substantial proportion of patients would remain underserved because they did not have sufficient data to run the algorithm. However, these susceptible patients were invisible. Second, clinical and operational stakeholders recognized that there was a significant social context missing from the data. Certain social support services were available, but without richer information about individual patient needs, the Population Rounding team was unable to address the potential barriers to PAD treatment. As described above in *Theme 2* (*Failure to Incorporate On-the-Ground Context*), the lack of social context heightened the role of PCPs, who had additional visibility into patients’ lives and highlighted a larger systemic need for improved documentation of social drivers. Although a more complex algorithm using natural language processing on clinicians’ notes was subsequently developed to address this challenge [19], the presented version of the algorithm used was chosen [21] for this study because of the ease of integration within IT systems. Finally, there were concerns among all stakeholder groups regarding the criteria used to identify impactable patients. Only patients with certain insurance plans were eligible for Population Rounding, and the pilot period focused on patients with upcoming PCP appointments. Patients at high risk for PAD who were not covered by a subset of insurance plans or patients without established access to primary care would not immediately benefit from ML-driven CDS. Taken together, these challenges present a stark reality for teams hoping to advance equity through the use of ML. The most susceptible patients, without robust data, without well-resourced insurance plans, and without regular access to care, are the least likely to benefit. These challenges will not be solved through technical innovations but will require structural changes in access to health care.

### Limitations

This study had several important limitations. First, this study did not include all relevant stakeholder groups affected by the ML-driven CDS for PAD. This decision was made because the early stage of integration meant that many of these stakeholders were yet to experience the effects of the PAD algorithm. Missing from our stakeholder cohort were patients whose care is impacted by the algorithm, payers, regulators, and policy makers. Second, although our study involved a broad group of stakeholders, there were limited clinical end users involved, and this was a single-site study, thus lacking external validation. Different sites may encounter different factors that influence the success of ML-driven CDS integration. Our study examined an ML algorithm that was internally built and is not currently planned for use on external sites; therefore, such an external validation was outside the scope of this study. Third, many organizations primarily adopt ML built by external EHR or third-party vendors, which are designed with a greater focus on spread across organizations and marketability [55]. By focusing on a custom-built algorithm within a single site, the findings of this study may overlook other factors related to the adoption of an externally built ML system, such as technical difficulties across different EHR systems, decentralized leadership, and a lack of trust by clinical users. Our Population Rounding workflow may not generalize to other workflow configurations; but we have attempted to maximize the generalizability of our data and findings through a detailed description of the tool, setting, and actors involved as well as an open and reflexive description of the researchers and their roles. Through this approach it may be that abstractions of the findings reported here, reinterpreted by local experts, will be more applicable to disparate contexts and ML-driven CDS than the PAD algorithm itself. Future studies are necessary to better delineate the variations between internally built versus externally procured systems to better guide integration. Fourth, our ML-driven CDS tool was well developed and previously validated in our study; thus, the results may not be generalizable to models that are technically poorly developed but may be insightful in translating successful in silico models into clinical care. Finally, our study was conducted during the first 4 weeks of the launch of the ML-driven CDS–informed Population Rounding process. Future work is needed to examine how quantitative results and the qualitative factors presented in this study change with time as the use of the algorithm evolves.

### Conclusions

Newly developed ML-driven CDS health care solutions continue to increase. Although many stakeholders are involved in the development, integration, and use of these systems, little is known about how these different stakeholder groups approach ML. In this qualitative study, technical, clinical, and operational stakeholders were interviewed to surface factors influencing successful integration of a ML-driven CDS for PAD. The following 3 themes emerged from the interviews: positive factors translating in silico performance to real-world efficacy, organizational factors and data structure factors affecting clinical impact, and challenges to advancing equity with ML. Findings highlight 3 potential best practices. First, health systems should invest in nurturing multistakeholder care delivery models that can be fertile ground for ML-driven CDS. Second, technical experts should be embedded among the clinical and operational stakeholders who drive the development and implementation of ML-driven CDS. Third, all team members should be mindful of the structural and environmental challenges that marginalize populations. Multidisciplinary perspectives must surface and address these challenges to enable ML-driven CDS to advance equity.

## Appendix

Multimedia Appendix 1 COREQ (Consolidated Criteria for Reporting Qualitative Research) checklist.

Multimedia Appendix 2 Interview guides.

Multimedia Appendix 3 Codebook with 5 inductive categories and 23 codes used in thematic analysis.

Multimedia Appendix 4 Duke institutional review board quality improvement checklist.

Multimedia Appendix 5 Participant information sheet.

## Abbreviations

CDS: clinical decision support
COREQ: Consolidated Criteria for Reporting Qualitative Research
EHR: electronic health record
IHI: Institute for Health Innovation
ML: machine learning
PAD: peripheral arterial disease
PCP: primary care provider
PHMO: Population Health Management Office

## Data Availability

The data sets analyzed during this study are not publicly available because of protection of interviewee identity owing to the large diversity of interviewee roles, despite interviews being anonymized. They may be available from the corresponding author on reasonable request.
