# Supplementary material for: Development and Integration of Machine Learning Algorithm to Identify Peripheral Arterial Disease: Multistakeholder Qualitative Study
Source: JMIR Form Res. 2023 Sep 21;7:e43963. doi: 10.2196/43963 (PMC10557008; doi:10.2196/43963)
Supplement: Multimedia Appendix 1 [file formative_v7i1e43963_app1.pdf]

No	Item	Guide questions/description	Location
**Domain 1: Research team and reflexivity**			
Personal Characteristics			
1.	Interviewer/facilitator	Which author/s conducted the interview or focus group?	Methods; Study Design and Data Collection
2.	Credentials	What were the researcher's credentials? *E.g. PhD, MD*	Authorship
3.	Occupation	What was their occupation at the time of the study?	Methods; Study Design and Data Collection
4.	Gender	Was the researcher male or female?	Authorship
5.	Experience and training	What experience or training did the researcher have?	Methods; Study Design and Data Collection
Relationship with participants			

No	Item	Guide questions/description	Location
6.	Relationship established	Was a relationship established prior to study commencement?	Methods; Study Design and Data Collection
7.	Participant knowledge of the interviewer	What did the participants know about the researcher? e*.g. personal goals, reasons for doing the research*	Methods; Study Design and Data Collection
8.	Interviewer characteristics	What characteristics were reported about the interviewer/facilitator? e.g. *Bias, assumptions, reasons and interests in the research topic*	Methods; Study Design and Data Collection
**Domain 2: study design**			
Theoretical framework			
9.	Methodological orientation and Theory	What methodological orientation was stated to underpin the study? *e.g. grounded theory, discourse analysis, ethnography, phenomenology, content analysis*	Methods; Study Design and Data Collection
Participant selection			

No	Item	Guide questions/description	Location
10.	Sampling	How were participants selected? *e.g. purposive, convenience, consecutive, snowball*	Methods; Study Design and Data Collection
11.	Method of approach	How were participants approached? e.*g. face-to-face, telephone, mail, email*	Methods; Study Design and Data Collection
12.	Sample size	How many participants were in the study?	Table 1
13.	Non-participation	How many people refused to participate or dropped out? Reasons?	Methods; Study Design and Data Collection
Setting			
14.	Setting of data collection	Where was the data collected? e.*g. home, clinic, workplace*	Methods; Study Design and Data Collection
15.	Presence of non-participants	Was anyone else present besides the participants and researchers?	Methods; Study Design and Data Collection

No	Item	Guide questions/description	Location
16.	Description of sample	What are the important characteristics of the sample? *e.g. demographic data, date*	Table 1
Data collection			
17.	Interview guide	Were questions, prompts, guides provided by the authors? Was it pilot tested?	Methods; Study Design and Data Collection
18.	Repeat interviews	Were repeat interviews carried out? If yes, how many?	Methods; Study Design and Data Collection
19.	Audio/visual recording	Did the research use audio or visual recording to collect the data?	Methods; Study Design and Data Collection
20.	Field notes	Were field notes made during and/or after the interview or focus group?	Methods; Study Design and Data Collection
21.	Duration	What was the duration of the interviews or focus group?	Methods; Study Design and Data Collection

No	Item	Guide questions/description	Location
22.	Data saturation	Was data saturation discussed?	Methods; Study Design and Data Collection
23.	Transcripts returned	Were transcripts returned to participants for comment and/or correction?	Data Analysis
**Domain 3: analysis and findings**			
Data analysis			
24.	Number of data coders	How many data coders coded the data?	Data Analysis
25.	Description of the coding tree	Did authors provide a description of the coding tree?	Supplementary material
26.	Derivation of themes	Were themes identified in advance or derived from the data?	Data Analysis
27.	Software	What software, if applicable, was used to manage the data?	Data Analysis
28.	Participant checking	Did participants provide feedback on the findings?	Data Analysis

No	Item	Guide questions/description	Location
Reporting			
29.	Quotations presented	Were participant quotations presented to illustrate the themes / findings? Was each quotation identified? e*.g. participant number*	Tables 2-5
30.	Data and findings consistent	Was there consistency between the data presented and the findings?	Results
31.	Clarity of major themes	Were major themes clearly presented in the findings?	Results
32.	Clarity of minor themes	Is there a description of diverse cases or discussion of minor themes?	Results
