# Supplementary material for: Development and Integration of Machine Learning Algorithm to Identify Peripheral Arterial Disease: Multistakeholder Qualitative Study
Source: JMIR Form Res. 2023 Sep 21;7:e43963. doi: 10.2196/43963 (PMC10557008; doi:10.2196/43963)
Supplement: Multimedia Appendix 2 [file formative_v7i1e43963_app2.pdf]

Clinician/End-User Interview Questions

1) **Key interests**
    a. What is your role at Duke? (4a.1)
    b. How has the PAD algorithm changed your clinical role? (4a.2, 4d.2)
    c. Who else do you work with to identify patients needing intervention and has the PAD algorithm altered those relationships? (4d.3)
2) **Daily work**
    a. What information do you seek before adopting any CDS (clinical decision support) tools into you clinical practice?
    b. Do you think the PAD algorithm is improving patient outcomes and do you feel able to check and evaluate its performance? (2b.2, 2b.1, 1a.3, 2b.3, 3b.4, 4a.3)
    c. Who else at Duke ought to be responsible for vetting algorithms like this and how? (6b.2, 6b.3, 5a.2)
    d. Do you think there's a need to improve specialty care for PAD patients at Duke, if so how? (1a.3, 5b.1, 2c.2, 4a.3, 4b.3)
    e. What have you found most challenging about the PAD or other algorithms?
    f. What is your favorite algorithm that you use in your work? What makes it your favorite?
3) **Key challenges**
    a. If you were using an even more accurate PAD algorithm, what do you think the impact on statin usage among PAD patients would be? (5)
    b. Are there other support or service changes that could be made to improve the algorithm's impact? (5)
    c. Is there anything you think limits your algorithm assisted recommendations for statin prescriptions translating to into patients actually taking them? (2c.1, 2c.2, 2f.2, 2f.3, 4a.3, 4b.3, 1b)
    d. Would you suggest any changes to the clinician's interface with the PAD algorithm or any other means of improving use by clinicians? (2a.1, 2c.1, 2c.3)
4) **Best practices**
    a. What algorithms have been most helpful for Duke clinicians to serve patients and why?
    b. What assurances help you feel confident in using an algorithm like PAD and where or who should they come from? (6b.2, 6b.3, 2b.2, 4a.2, 4d.3)
    c. How do you think the patient value of an algorithm like PAD could be maximised within Duke? (2f)
    d. How would you define success for this model? (2f)
5) **Closing**
    a. Is there anyone else you think we need to speak to who isn't currently on our list?
    b. Anything to add that you would do differently when using algorithms for decision support?
    c. Based on your current engagement in PAD project how would you rate the ease of development and deployment of algorithm-based solutions into clinical practise?

PHMO Interview Questions

1) **Key Interests:**
    a. What is your role at Duke?
    b. How will you be engaged with this PAD project?
    c. What should this project accomplish for Duke PHMO?
2) **Daily Work:**
    a. Do you believe that PAD is being adequately diagnosed among PHMO patients? Why or why not?
        i. Do you believe there is an opportunity for an algorithm and novel workflow to improve care for PHMO PAD patients?
    b. When considering a new algorithm or workflow to improve PAD care, are there subgroups of patients that you pay special attention to?
    c. How do you think this project can successfully create value for PHMO?
    d. What do you see as the most important ways that PHMO can better serve adults with PAD?
    e. Aside from the new algorithm and workflow, are there any complementary programs or services that are needed or that PHMO is putting in place to better serve patients with PAD?
3) **Key Challenges:**
    a. What have been your biggest challenges in using screening algorithms?
    b. What information would you want to see to feel confident using an algorithm like the PAD algorithm?
    c. Improving primary care statin prescriptions to PAD patients is one of main success criteria for the project. How do you think the project will affect this outcome?
        i. What other communication channels or personnel can help support the model achieve that outcome?
    d. What concerns do you have about implementing this model?
    e. Do you think it is sustainable beyond DIHI's involvement for start-up?
    f. How would you define success for this PAD algorithm solution?
4) **Closing**
    a. Is there anyone else you think we need to speak to who isn't currently on our list?
    b. What else would you like to add which can further support the successful implementation of this project?

Technical Staff Interview Questions

1) Open Specific:

- What is your role at Duke / what department are you in?
  - How does this role support clinicians that care for PAD patients?
- How do you engage stakeholders to determine relevant patient information when developing a model to identify a patient with Peripheral Artery Disease?

2) Daily Work:

- Where do you see the most common failure of algorithms identifying high risk patients?
- How are you able to assess if the model is performing appropriately?
- How are you able to assess if the model is identifying all patient populations equally?
- How would new data changes or practice guidelines be updated in the model?
- For end-users with less technological background, what strategies can be used to strengthen confidence and certainty in model outputs?
- What would be potential challenges for other clinical teams that want to implement this model in practice?

3) Key Challenges:

- What concerns do you have about this project?
- What should this project accomplish for the programming team?
- What is your biggest takeaway when developing this model that can be applied to building future similar algorithms?

4) Closing

- Is there anyone else you think we need to speak to who isn't currently on our list?
- How would you like to be involved in the rest of the project?
