# Supplementary material for: Development and Integration of Machine Learning Algorithm to Identify Peripheral Arterial Disease: Multistakeholder Qualitative Study
Source: JMIR Form Res. 2023 Sep 21;7:e43963. doi: 10.2196/43963 (PMC10557008; doi:10.2196/43963)
Supplement: Multimedia Appendix 3 [file formative_v7i1e43963_app3.docx]

# Categories and codes

## Healthcare workforce

- 1. Available skill sets and disciplines
  2. Capacity and resourcing
  3. Alignment with current practice
  4. Interdisciplinary dialogue and barriers

## Model Factors

- 1. Data Ingestion (process)
  2. Data changes
  3. Data Ingestion (quality)
  4. Data transparency
  5. Data Bias and Equity
  6. Nature of data output
  7. Model Training and evaluation
  8. Model Explainability

## Model Intervention/Implementation

- 1. Nature of intervention output
  2. Barriers to model interpretability

## Model Placement

- 1. Alignment with current practice
  2. Implementation barriers
  3. Relationship with adopters

## Organisations

- 1. Supportive leadership
  2. Organizational structure
  3. Innovative and reflexive culture
  4. Resources
  5. Motives and success criteria
  6. Interdisciplinary dialogue and barriers
