# Supplementary material for: Development and Integration of Machine Learning Algorithm to Identify Peripheral Arterial Disease: Multistakeholder Qualitative Study
Source: JMIR Form Res. 2023 Sep 21;7:e43963. doi: 10.2196/43963 (PMC10557008; doi:10.2196/43963)
Supplement: Multimedia Appendix 4 [file formative_v7i1e43963_app4.pdf]

DUKE UNIVERSITY HEALTH SYSTEM
Human Research Protection Program

QUALITY IMPROVEMENT ACTIVITIES IN HEALTH CARE VERSUS RESEARCH

4/14/2021

Policy Scope

In general, a quality improvement (QI) project does not need to be submitted to the IRB. An IRB submission is needed by Duke students/trainees and faculty who are conducting quality improvement projects that require an authoritative determination of whether an activity does or does not meet the definition of research with humans. An authoritative determination might be required by Departmental policy or as a condition of a training program or by a journal or conference prior to acceptance of a health care related manuscript for publication or presentation. When Investigators and staff are seeking an authoritative determination, these proposed QI activities should be submitted through iRIS for evaluation by the IRB. Investigators should request an exemption from further IRB review, resulting in a greatly shortened iRIS application form (See the QI application template on the IRB website). Investigators and DUHS staff may always consult with a DUHS IRB Chair or the DUHS IRB Executive Director to discuss whether an activity does or does not meet the definition of research with humans and may require submission to the IRB. A checklist is found on p.4 that may be helpful in determining whether a proposed activity is a QI project and does not involve human subjects research. The IRB cannot issue retroactive approval of an activity that is conducted as a QI project and is later determined to be human research.

Research is defined in 45 CFR 46.102 and 45 CFR 164.501 as "a systematic investigation, including research development, testing and evaluation, designed to develop or contribute to generalizable knowledge."[1] Quality improvement (QI) in health care, unlike research, focuses on translating existing knowledge from research into clinical practice to improve the quality of health care for individuals and populations. The key difference between these two concepts is that research studies are intended to create new knowledge that can be generalizable to other populations and settings, while QI in health care uses existing knowledge to improve health care outcomes within a local health care institution or setting.[2-3] Health care institutions have evolved into systems that collect, aggregate, analyze and learn from patient-level data where clinicians make evidence-based practice decisions guided by general knowledge produced from structured learning.[4] The new knowledge generated from research or the collection of evidence-based practices often requires further evaluation when applied in a specific health-care setting. QI activities provide important information on the application of existing knowledge and changes that may be needed to achieve the best possible clinical outcomes.[3]

When an activity involving the inclusion of people is intended to evaluate an existing practice and attempt to improve it based upon existing knowledge, and if the data from the evaluation is not intended to be applied to populations other than the population under study, then the DUHS IRB would not classify this activity as research, and the activity would not be subject to the DHHS human research regulations. Likewise, the intent to publish is an insufficient criterion for determining whether a QI activity involves research.[5] Even planning to publish an account of a QI project does not necessarily mean that the project fits the definition of research. People seek to publish descriptions of non-research activities for a variety of reasons, including, for example, if they believe others may be interested in what worked at another institution. A major priority for the National Quality Strategy[3] is to develop and share methods for data collection, measurement, and reporting that support QI measurement and improvement efforts of both public and private sector stakeholders at the national and community level.* Dissemination of QI efforts will require timely publication and sharing of information to create awareness of lessons

learned, as well as what QI projects work well within each other's institutions.+ When an activity involves the inclusion of people to test a new, modified, or previously untested intervention, service, or program for which there is insufficient evidence to determine whether it is safe and/or effective, this is research involving humans, and it is subject to IRB review and approval. A comparative intervention study examining two evidence-based methods, with people randomized between the two methods to determine which is better, is also regarded as research involving humans.

The projects described below are examples of how evidence-based practice change implementation may be conducted without involving human subjects in research. Additional examples of quality improvement projects that are not identified as research involving human subjects are found on the U.S. Department of Health & Human Services website.[6]

1) DUHS implements an evidence-based approach to reducing pharmacy prescription errors and collects prescription practices by chart review. Adherence to this approach and medication error rates are evaluated after implementation. The team plans to submit to a journal that requires IRB review and the project is submitted to the IRB for an authoritative determination that the planned activity does not meet the definition of research with humans.

2) A graduate student project will evaluate the effect of standardizing care for patients presenting to the Duke Hospital Emergency Room with diabetic ketoacidosis using the evidence-based guideline published by the American Diabetes Association. A DEDUCE search identifies patients admitted for diabetic ketoacidosis a year before the evidence-based care guideline was implemented and also for patients treated a year later in compliance with the guideline. Outcome measures include provider guideline adherence as well as clinically specific indicators that measure timely and efficient reversal of ketoacidosis. As required by the student's program, the project is submitted to the IRB for an authoritative determination that the activity does not meet the definition of research with humans.

3) The staff of an adult oncology clinic cares for patients receiving chemotherapy that commonly causes severe mucositis. The staff members implement a widely accepted oral care assessment tool as part of routine standard of care. An evidence-based training program on how to use the oral assessment tool is provided to the patient care team. A chart review a month later is used to evaluate whether a change in practice has occurred, measured by the number or oral care assessments performed and whether these assessments were performed with appropriate patients.

For projects not involving humans in research, but involving the recording of identifiable private information, standard privacy and confidentiality considerations apply. Surveillance for disease, such as occurs through the DUHS Infection Control Program or the Duke Tumor Registry, typically does not meet the definition of research.[6] Even when identifiable private information is provided to State or federal agencies as a part of mandated public health surveillance programs, DUHS, its employees and agents are not engaged in research with humans. Such activities do not require DUHS IRB approval.

---

*To provide further perspective on the importance of QI in health care, in March 2011, the U.S. Department of Health and Human Services (DHHS) released the inaugural report to Congress on the National Strategy for Quality Improvement in Healthcare.[3] This National Quality Strategy focuses on three QI aims:

- **Better Care:** Improve the overall quality of care, by making health care more patient-centered, reliable, accessible, and safe.
- **Healthy People/Healthy Communities:** Improve the health of the U.S. population by supporting proven interventions to address behavioral, social, and environmental determinants of health in addition to delivering higher- quality care.
- **Affordable Care:** Reduce the cost of quality health care for individuals, families, employers, and government.

+The Hastings Center Report stresses the importance of learning health care systems committed to carrying out quality improvement activities. As health care systems continue to evolve, it is evident that clinical practice cannot be of the highest quality if it is independent of its connection with ongoing, systematic learning.[4,7] Learning healthcare systems, described in the Hastings Center Report, view clinical practice as an ongoing source of data to be used for continuously changing and improving patient care.

References

1. Code of Federal Regulations. Department of Health and Human Services. Effective July 19, 2018. https://www.hhs.gov/ohrp/regulations-and-policy/regulations/45-cfr-46/index.html

2. Institute of Medicine. Crossing the Quality Chasm: A New Health System for the 21st Century. Washington, DC: National Academies Press, 2001.

3. National Strategy for Quality Improvement in Health Care. Annual Progress Report to Congress. U.S. Department of Health and Human Services, Washington, DC, 2012.

4. Kass, N.E., Faden, R.R., Goodman, S.N., et al. (2013). The research-treatment distinction: A problematic approach for determining which activities should have ethical oversight. Hastings Center Report, Jan-Feb, S4-S15.

5. OHRP Quality Improvement Activities Frequently Asked Questions. U.S. Department of Health and Human Services. https://www.hhs.gov/ohrp/regulations-and-policy/guidance/faq/quality-improvement-activities/index.html

6. *OHRP Guidance on Research Involving Coded Private Information or Biological Specimens*, October 2008; available at http://www.hhs.gov/ohrp/policy/cdebiol.pdf.

7. Solomon M.Z., Bonham, A.C. (2013). Ethical oversight of research on patient care. Hastings Center Report, Jan-Feb, S2-S3.

Previous Version Date(s): 6/1/2007, 3/9/2009, 4/30/2013, 3/3/2016

Duke University Institutional Review Board
Quality Improvement or Research Checklist*

In general, a quality improvement (QI) project does not require IRB review and approval because it is not research that is subject to the federal human subjects protection regulations. The following questions may be helpful in determining whether a proposed activity is a QI project and does not involve human subjects research. If all of the questions below can be answered as a Yes, **IRB review is not required.** If the answer to any of these questions is NO, please consult with the IRB for assistance since IRB review may be required.** An investigator or staff member may also request an authoritative determination from the IRB to confirm or assist with determining if an activity is a quality improvement project. To obtain this determination, the project should be submitted through iRIS using the QI template summary.***

Project Description	YES	NO
**Purpose**<br>Is the activity intended to improve the process/delivery of care while decreasing inefficiencies within a specific health care setting?		
**Scope**<br>Is the activity intended to evaluate current practice and/or attempt to improve it based upon existing knowledge?		
**Evidence**<br>Is there sufficient existing evidence to support implementing this activity to create practice change?		
**Clinicians/Staff**<br>Is the activity conducted by clinicians and staff who provide care or are responsible for the practice change in the institutions where the activity will take place?		
**Methods**<br>Are the methods for the activity flexible and include approaches to evaluate rapid and incremental changes?		
**Sample/Population**<br>Will the activity involve a sample of the population (patients/participants) ordinarily seen in the institution where the activity will take place?		
**Consent**<br>Will the planned activity only require consent that is already obtained in clinical practice, and could the activity be considered part of the usual care?		
**Benefits**<br>Will future patients/participants at the institution where the planned activity will be implemented potentially benefit from the project?		
**Risk**<br>Is the risk to patients/participants no greater than what is involved in the care they are already receiving **OR** can participating in the activity be considered acceptable or ordinarily expected when practice changes are implemented within a health care environment?		

***Adapted with permission from the Yale University IRB, March 2013**
****An inquiry can be made by email to the DUHS IRB by going to the IRB website at https://irb.duhs.duke.edu/. Click on "Contact Us."**
***** The QI Summary Template is found on the IRB website under Forms, or use the website search bar to search for "QI Summary Template."**
