# Supplementary material for: Development and Integration of Machine Learning Algorithm to Identify Peripheral Arterial Disease: Multistakeholder Qualitative Study
Source: JMIR Form Res. 2023 Sep 21;7:e43963. doi: 10.2196/43963 (PMC10557008; doi:10.2196/43963)
Supplement: Multimedia Appendix 5 [file formative_v7i1e43963_app5.pdf]

Project Information Sheet
Improving Equity and Value of Peripheral Artery Disease (PAD) with AI

My name is Sabrina Wang and I am a current MD/MPH student at Duke University SOM and UNC Gillings. I am working with the Duke Institution for Health Innovation (DIHI) to assess the development and integration of an augmented-intelligence (AI) algorithm to improve identification, treatment, and overall health outcomes of patients with PAD. This project is part of a broader effort to ensure the rigorous audit, evaluation, and monitoring of AI tools used in clinical care. This study will assess procurement, integration, and monitoring of the AI algorithm by healthcare providers, organizational leaders, and others involved in the operationalization and maintenance of the tool. We hope to further improve the development and implementation of future AI tools.

If you agree to the study, I will ask you to complete an interview to understand your views on how this AI tool can be utilized in practice and what further information would be useful for implementation. We hope this work improves the quality, equity, and value of care for patients with PAD, and your honest input is important to the project success.

1) Participation in the study is voluntary and your decision to participate will be kept confidential.
2) You have the right not to answer any question or to stop an interview at any time.
3) Your confidentiality will be protected:
    a) Notes are securely stored on a password protected, encrypted Duke computer and shared via a secure cloud (Box)
    b) Results and themes will be studied collectively. Any quotes will be nameless and will not include information, such as role, that would identify the individual.
4) Recorded interviews: Conversations will only be recorded if you agree. These would help us to have more thorough analysis that written notes may not be able to convey.
    a) You have the right to revoke recording permission at any time, even if permission was granted prior.
    b) Recordings will similarly be stored on a password protected, encrypted Duke computer and shared via a secure cloud (Box)

Should you have any questions regarding the interview process or your rights as a research subject, feel free to contact me with any questions at smw98@duke.edu or 262-271-0548.
